# Supplementary material for: Iterative improvement in the automatic modular design of robot swarms
Source: PeerJ Comput Sci. 2020 Dec 7;6:e322. doi: 10.7717/peerj-cs.322 (PMC7924708; doi:10.7717/peerj-cs.322)
Supplement: Supplemental Information 3 [file peerj-cs-06-322-s003.zip › argos3/doc/api/standalone/a00623_source.html]

ARGoS: plugins/simulator/physics\_engines/pointmass3d/pointmass3d\_engine.cpp Source File


- Main Page
- Related Pages
- Namespaces
- Classes
- Files

- File List
- File Members

# plugins/simulator/physics\_engines/pointmass3d/pointmass3d\_engine.cpp

Go to the documentation of this file.

```
00001 
00007 #include "pointmass3d_engine.h"
00008 #include "pointmass3d_model.h"
00009 #include <argos3/core/utility/logging/argos_log.h>
00010 #include <argos3/core/utility/configuration/argos_configuration.h>
00011 
00012 namespace argos {
00013 
00014    /****************************************/
00015    /****************************************/
00016 
00017    CPointMass3DEngine::CPointMass3DEngine() :
00018       m_fGravity(-9.81f) {
00019    }
00020 
00021    /****************************************/
00022    /****************************************/
00023 
00024    CPointMass3DEngine::~CPointMass3DEngine() {
00025    }
00026 
00027    /****************************************/
00028    /****************************************/
00029 
00030    void CPointMass3DEngine::Init(TConfigurationNode& t_tree) {
00031       /* Init parent */
00032       CPhysicsEngine::Init(t_tree);
00033       /* Set gravity */
00034       GetNodeAttributeOrDefault(t_tree, "gravity", m_fGravity, m_fGravity);
00035    }
00036 
00037    /****************************************/
00038    /****************************************/
00039 
00040    void CPointMass3DEngine::Reset() {
00041       for(CPointMass3DModel::TMap::iterator it = m_tPhysicsModels.begin();
00042           it != m_tPhysicsModels.end(); ++it) {
00043          it->second->Reset();
00044       }
00045    }
00046 
00047    /****************************************/
00048    /****************************************/
00049 
00050    void CPointMass3DEngine::Destroy() {
00051       /* Empty the physics entity map */
00052       for(CPointMass3DModel::TMap::iterator it = m_tPhysicsModels.begin();
00053           it != m_tPhysicsModels.end(); ++it) {
00054          delete it->second;
00055       }
00056       m_tPhysicsModels.clear();
00057    }
00058 
00059    /****************************************/
00060    /****************************************/
00061 
00062    void CPointMass3DEngine::Update() {
00063       /* Update the physics state from the entities */
00064       for(CPointMass3DModel::TMap::iterator it = m_tPhysicsModels.begin();
00065           it != m_tPhysicsModels.end(); ++it) {
00066          it->second->UpdateFromEntityStatus();
00067       }
00068       for(size_t i = 0; i < GetIterations(); ++i) {
00069          /* Perform the step */
00070          for(CPointMass3DModel::TMap::iterator it = m_tPhysicsModels.begin();
00071              it != m_tPhysicsModels.end(); ++it) {
00072             it->second->Step();
00073          }
00074       }
00075       /* Update the simulated space */
00076       for(CPointMass3DModel::TMap::iterator it = m_tPhysicsModels.begin();
00077           it != m_tPhysicsModels.end(); ++it) {
00078          it->second->UpdateEntityStatus();
00079       }
00080    }
00081 
00082    /****************************************/
00083    /****************************************/
00084 
00085    size_t CPointMass3DEngine::GetNumPhysicsModels() {
00086       return m_tPhysicsModels.size();
00087    }
00088 
00089    /****************************************/
00090    /****************************************/
00091 
00092    bool CPointMass3DEngine::AddEntity(CEntity& c_entity) {
00093       SOperationOutcome cOutcome =
00094          CallEntityOperation<CPointMass3DOperationAddEntity, CPointMass3DEngine, SOperationOutcome>
00095          (*this, c_entity);
00096       return cOutcome.Value;
00097    }
00098 
00099    /****************************************/
00100    /****************************************/
00101 
00102    bool CPointMass3DEngine::RemoveEntity(CEntity& c_entity) {
00103       SOperationOutcome cOutcome =
00104          CallEntityOperation<CPointMass3DOperationRemoveEntity, CPointMass3DEngine, SOperationOutcome>
00105          (*this, c_entity);
00106       return cOutcome.Value;
00107    }
00108 
00109    /****************************************/
00110    /****************************************/
00111 
00112    bool CPointMass3DEngine::IsPointContained(const CVector3& c_point) {
00113       return true;
00114    }
00115 
00116    /****************************************/
00117    /****************************************/
00118 
00119    bool CPointMass3DEngine::IsEntityTransferNeeded() const {
00120       return false;
00121    }
00122 
00123    /****************************************/
00124    /****************************************/
00125 
00126    void CPointMass3DEngine::TransferEntities() {
00127    }
00128 
00129    /****************************************/
00130    /****************************************/
00131 
00132    void CPointMass3DEngine::CheckIntersectionWithRay(TEmbodiedEntityIntersectionData& t_data,
00133                                                      const CRay3& c_ray) const {
00134       Real fTOnRay;
00135       for(CPointMass3DModel::TMap::const_iterator it = m_tPhysicsModels.begin();
00136           it != m_tPhysicsModels.end();
00137           ++it) {
00138          if(it->second->CheckIntersectionWithRay(fTOnRay, c_ray)) {
00139             t_data.push_back(
00140                SEmbodiedEntityIntersectionItem(
00141                   &it->second->GetEmbodiedEntity(),
00142                   fTOnRay));
00143          }
00144       }
00145    }
00146 
00147    /****************************************/
00148    /****************************************/
00149 
00150    void CPointMass3DEngine::AddPhysicsModel(const std::string& str_id,
00151                                             CPointMass3DModel& c_model) {
00152       m_tPhysicsModels[str_id] = &c_model;
00153    }
00154 
00155    /****************************************/
00156    /****************************************/
00157 
00158    void CPointMass3DEngine::RemovePhysicsModel(const std::string& str_id) {
00159       CPointMass3DModel::TMap::iterator it = m_tPhysicsModels.find(str_id);
00160       if(it != m_tPhysicsModels.end()) {
00161          delete it->second;
00162          m_tPhysicsModels.erase(it);
00163       }
00164       else {
00165          THROW_ARGOSEXCEPTION("PointMass3D model id \"" << str_id << "\" not found in point-mass 3D engine \"" << GetId() << "\"");
00166       }
00167    }
00168 
00169    /****************************************/
00170    /****************************************/
00171 
00172    REGISTER_PHYSICS_ENGINE(CPointMass3DEngine,
00173                            "pointmass3d",
00174                            "Carlo Pinciroli [ilpincy@gmail.com]",
00175                            "1.0",
00176                            "A 3D point-mass physics engine.",
00177                            "This physics engine is a 3D point-mass engine.\n\n"
00178                            "REQUIRED XML CONFIGURATION\n\n"
00179                            "  <physics_engines>\n"
00180                            "    ...\n"
00181                            "    <pointmass3d id=\"pm3d\" />\n"
00182                            "    ...\n"
00183                            "  </physics_engines>\n\n"
00184                            "The 'id' attribute is necessary and must be unique among the physics engines.\n"
00185                            "If two engines share the same id, initialization aborts.\n\n"
00186                            "OPTIONAL XML CONFIGURATION\n\n"
00187                            "None for the time being.\n\n"
00188                            ,
00189                            "Under development"
00190       );
00191 
00192 }
```

---

Generated on 10 Jul 2018 for ARGoS by 
 1.6.1
